# A pilot randomised trial comparing individualised physiotherapy versus shockwave therapy for proximal hamstring tendinopathy: a protocol

**DOI:** 10.1186/s40634-023-00615-x

**Published:** 2023-05-25

**Authors:** Aidan Lindsay Fenner Rich, Jillianne Leigh Cook, Andrew John Hahne, Jon Joseph Ford

**Affiliations:** 1 School of Allied Health, Human Services and Sport, La Trobe University, Plenty Road, Bundoora, VIC 3086 Australia; 2 Advance Healthcare, 157 Scoresby Road, Boronia, VIC 3155 Australia; 3 Lifecare Ashburton Sports Medicine, 330 High Street, Ashburton, VIC 3147 Australia

## Abstract

**Purpose:**

Proximal hamstring tendinopathy (PHT) presents as localised lower buttock pain with tasks such as squatting and sitting. It is a condition that occurs at all ages and levels of sporting participation and can cause disability with sport, work, and activities of daily living.

This paper details a pilot trial protocol for investigating the effectiveness of individualised physiotherapy compared to extracorporeal shockwave therapy (ESWT) on pain and strength in people with PHT.

**Methods:**

The study is an assessor-blinded, pilot randomised controlled trial (RCT). One hundred participants with PHT will be recruited from the local community and sporting clubs. Participants will be randomised to receive six sessions of either individualised physiotherapy or ESWT, with both groups also receiving standardised education and advice.

Primary outcomes will be global rating of change on a 7-point Likert scale, and the Victorian Institute of Sport—Hamstring (VISA-H) scale, measured at 0, 4, 12, 26 and 52 weeks. Secondary outcomes will include sitting tolerance, the modified Physical Activity Level Scale, eccentric hamstring strength, modified Tampa scale for kinesiophobia, the Örebro Musculoskeletal Pain Screening Questionnaire Short Form (ÖMPSQ-SF), Numerical Pain Rating Scale (NPRS) for average and worst pain, participant adherence, the Pain Catastrophizing scale, satisfaction scores, and quality of life. Data will be analysed on an intention to treat basis, with between-group effects estimated using linear mixed models for continuous data and Mann Whitney U tests for ordinal data.

**Conclusions:**

This pilot RCT will compare individualised physiotherapy versus ESWT for PHT. The trial will determine feasibility and estimated treatment effects to inform a definitive trial in the future.

**Trial registration:**

The trial has been prospectively registered with the Australia & New Zealand Clinical Trials Registry (ACTRN12621000846820), registered 1 July 2021, https://www.anzctr.org.au/Trial/Registration/TrialReview.aspx?id=373085

**Supplementary Information:**

The online version contains supplementary material available at 10.1186/s40634-023-00615-x.

## Background

Proximal hamstring tendinopathy (PHT) affects active people, particularly those participating in running, lunging, or kicking sports. This condition was initially described in 1988 [1] as ‘hamstring syndrome’ and typically is associated with focal lower buttock pain, aggravated by prolonged sitting, running/walking, lunging and squatting [2–4].

Tendinopathy is attributed to acute and/or chronic overloading or unloading of the tendon. Tendon loading comprises of tensile and compressive components. Acute loading can result in reactive tendinopathy [5] that manifests as tendon swelling due to increased large proteoglycan content, but without collagen disruption. Chronic overloading can lead to ‘disrepair’ or ‘degenerative’ [5] tendinopathies, with collagen and tendon matrix disorganisation.

While the primary function of tendons appears to be to transmit tensile loads, compressive forces have been shown to be a contributor to adaptive changes of the tendon matrix and development of tendon pathology [6]. Compressive load in PHT generally occurs at the ischial tuberosity during activities involving deeper ranges of hip flexion, such as squatting, lunging, kicking and hamstring stretching, or with direct compression in sitting. Activities involving compressive loading are commonly provocative in PHT [3, 4]. Surgical [7] and imaging [8] studies show the location of tendinopathic change in PHT is adjacent to the ischial tuberosity insertion in PHT suggesting compression as a key factor in the development of this condition.

There is limited research on the diagnostic criteria for PHT and an absence of a diagnostic gold/reference standard suitable for robust studies on diagnostic accuracy. A study [9] on diagnostic accuracy using MRI as the reference standard investigated three different stretching tests. However, the conclusions of this research are limited because of the high prevalence of abnormal proximal hamstring findings in asymptomatic people [10].

Based on biological plausibility, there is general consensus on the importance of pain on tensile and/or compressive loading of the tendon (such as in muscle contraction, functional tasks, stretching or sitting [1, 3, 4, 11]), and pain location over the proximal tendon as clinical features indicative of PHT [12–16]. An additional potentially relevant clinical feature is a history of increased tendon load precipitating onset of symptoms [3, 11].

A number of treatment options have been proposed for PHT. Traditionally, conservative care has included load management, graded rehabilitation/exercise, selective use of oral non-steroidal anti-inflammatory drugs, and manual therapy techniques [3, 4]. Injection therapies such as platelet rich plasma, autologous blood injection and corticosteroid injection have also been investigated. There is an absence of high-quality research demonstrating effectiveness of these approaches in PHT [8, 17–21].

Extracorporeal shockwave therapy (ESWT) is a non-invasive and commonly used treatment that has demonstrated effectiveness in a variety of tendinopathies [22–26]. The mechanisms underpinning the effects of ESWT in tendinopathy are uncertain but reduced pain pressure threshold has been demonstrated in normal Achilles tendons [27, 28]. Changes in tendon collagen structure in normal animal tendons [27, 29] have also been demonstrated in response to ESWT. An RCT comparing ESWT to conservative care for PHT, [2] showed superior results on pain and function at 3-month follow-up, although trial limitations included the conservative program not reflecting successful protocols in other tendinopathy trials, [30] and being generic rather than individualised to participant presentation [31].

Recent narrative reviews and mechanistic papers support the use of individualised rehabilitation for PHT. Key recommendations include progressive strengthening exercises, graduated reintroduction of compressive loads, restoration of tendon energy storage and release capacity and return to normal activity [3, 11]. Progressive strengthening programs have demonstrated improvement in pain, disability and function in other lower limb tendinopathies [32–45] however this approach has not been evaluated for PHT particularly when individualised to the participant presentation; an approach common in other musculoskeletal research [46–48].

Given the shortcomings of the literature as described above, there is need for further evaluation on the effectiveness of individualised physiotherapy for people with clinical features indicative of PHT. The aim of this paper is to describe the design of a pilot RCT comparing the effectiveness of individualised physiotherapy (‘PHYSIOTHERAPY’) with ESWT (‘SHOCKWAVE’) on pain, strength and function for people with PHT. The objectives for this trial are to assess feasibility of recruitment and follow up of people with PHT for a future effectiveness trial, and to measure outcome domains (including for global rating of change, pain, function and sporting participation) in participants with PHT.

## Methods/design

### Study design

**Fig. 1:**
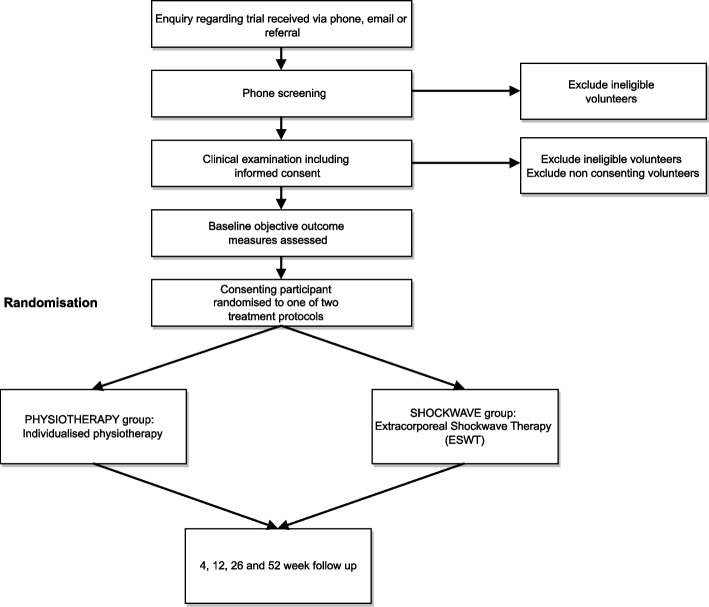
Study design

This trial will be an assessor-blinded, pilot RCT comparing two interventions: PHYSIOTHERAPY and SHOCKWAVE (Fig. 1).

### Patient and public involvement

There was no formal involvement of participants in the study design however two of the researchers treat people with PHT providing indirect patient input into the development of the study protocols.

### Ethics and registration

Ethical approval has been received from the La Trobe University Human Ethics Committee (HEC21049). The trial has been prospectively registered with the Australia & New Zealand Clinical Trials Registry (ACTRN12621000846820).

### Setting

Treatment will be conducted at private physiotherapy practices throughout Victoria, Australia.

### Eligibility and screening

Participants will be sought via referrals from orthopaedic surgeons, physiotherapists, medical practitioners and through direct public advertising. Personal correspondence, group presentations, formal meetings and trial information sheets will be used to inform potential referrers. This will be supplemented with public advertising about the trial through social media and print media.

**Fig. 2:**
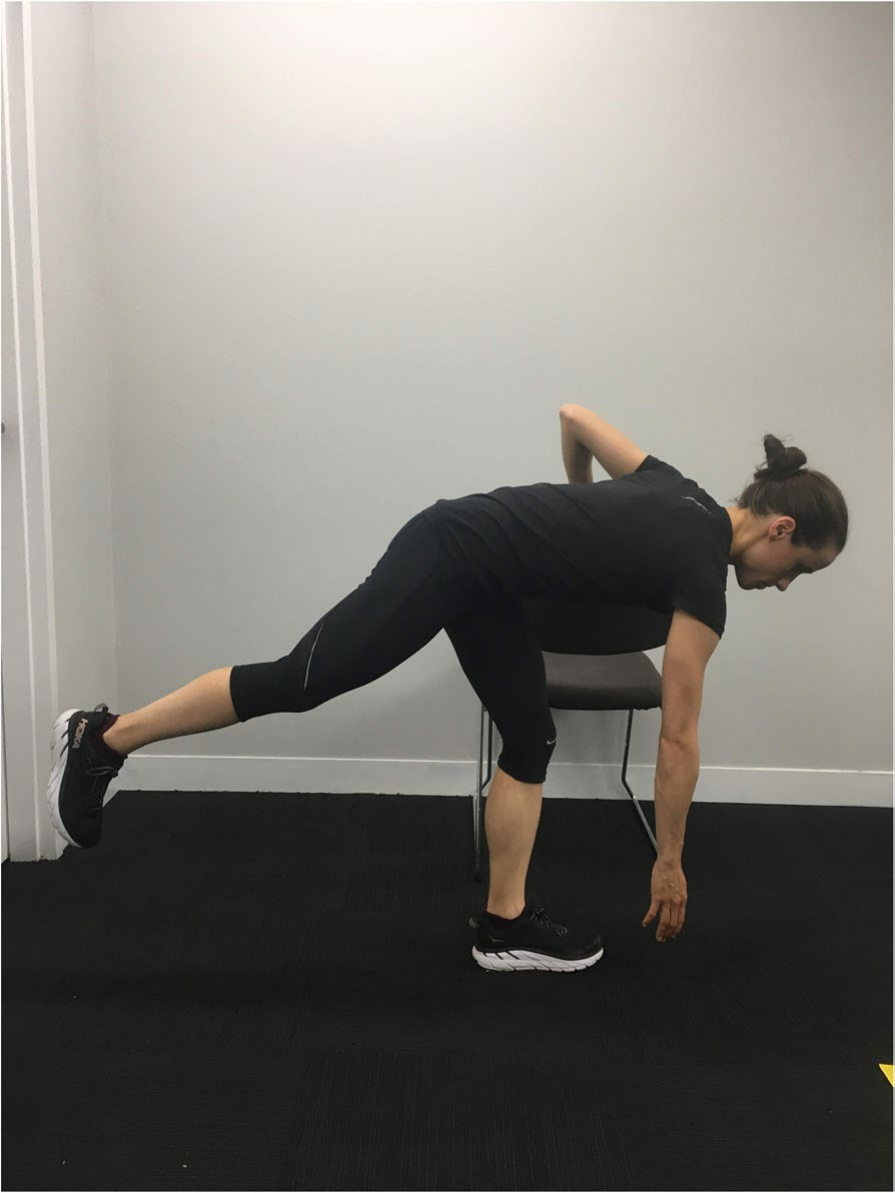
Single leg arabesque

**Fig. 3:**
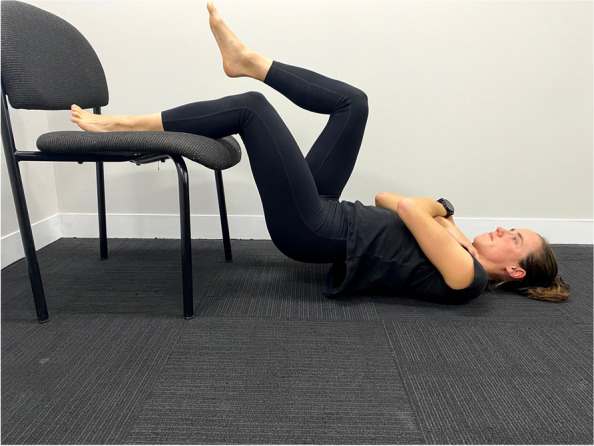
Single leg hamstring bridge

Participants with clinical features of PHT, aged from 18–65, will be recruited. Initial eligibility screening will occur via telephone. The clinical examination with the lead researcher will confirm eligibility and provide descriptive information on the baseline characteristics of participants. Participants will need to have clinical features indicative of PHT including: localised ischial tuberosity region pain, a history of increased tendon load precipitating onset of symptoms, and reproduction of pain on three or more loading/compressive tests (Figs. 2 and 3, Table 1). All tests have biological plausibility for providing high compressive ± tensile load to the tendon and have consensus in the literature as being beneficial in diagnosing PHT [3, 4, 11]. If eligibility is confirmed at clinical examination, the volunteer will be sent the study plain language statement and consent form that explains the study requirements, procedures, and time commitments.

**Table 1:** Eligibility criteria

**Inclusion Criteria**
* Initial Phone Screening*
1. Reports of relatively localised (defined as an area smaller than a tennis ball) ischial tuberosity region pain [3] of gradual onset and at least 3 months in duration
2. Willingness to participate in six sessions of intervention over a 12-week period
3. Age between 18 and 65 inclusive
4. Fluency in English sufficient to complete questionnaires and to enable understanding to the intervention
5. Agreeing to refrain from other interventions for the treatment period of the trial, aside from consultation with medical practitioners, and medication
* Clinical examination screening*
6. A history of increased tendon load precipitating the onset of symptoms determined based on clinical interview
7. Reproduction of ischial tuberosity region pain with three or more of the following loading/compressive tests:
• Single-leg arabesque (Fig. 2)
• Supine single leg bridge with heel on standardised height platform (bent knee) (Fig. 3)
• Self-reported PHT symptoms with prolonged sitting < 30 min
• Modified bent-knee hamstring stretch test [9]
**Exclusion Criteria**
* Initial Phone Screening*
1. Previous surgery to the hamstring complex, as we wish to study treatment effects independent to the effects of surgical procedures
2. Previous injection to the hamstring tendon within the previous 6 weeks, as we wish to study treatment effects independent to the effects of injections
3. Treatment with ESWT for PHT in the last 3 months, [26] as this may influence progress during the trial
4. Contraindications to receiving ESWT [49]
5. Current pregnancy, or recent childbirth (within 6 months) as this could impair ability to undertake testing and intervention
6. Diagnosis with autoimmune disease as these conditions may have a negative impact on tendon
7. Already received more than two sessions of physiotherapy with any of the trial physiotherapists prior to enrolment, as these therapists are likely to use components of the trial treatment protocol on their clinical caseload
8. An active compensation claim for the injury, as this may influence the response to treatment [50]
9. Planned absence for a period of > 2 weeks during the treatment period (such as extended holiday)
* Clinical examination screening*
10. Pain that is predominantly due to lumbar dysfunction including lumbar spine radiculopathy, [51] or lumbar spine somatic referral (Additional file 1)
11. Pain that is reasoned from clinical examination to be predominantly due to other structures or conditions, including sciatic nerve entrapment, ischiofemoral impingement, hip joint, local sciatic nerve irritation, and adductor magnus tendinopathy (Additional file 1)

### Randomisation and allocation

Following provision of written consent, participants will be randomised into one of two intervention groups: PHYSIOTHERAPY or SHOCKWAVE. A researcher located remotely at La Trobe University who will have no contact with trial participants will prepare a randomisation schedule ahead of time. The randomisation sequence will be generated electronically by an online randomisation program that incorporates random block sizes. Randomisation will be stratified for age (< 50 years of age vs >  = 50 years of age), as systemic factors associated with menopause may influence response to some components of treatment [52–55]. Concealed allocation of participants in accordance with the randomisation schedule will be undertaken by the same researcher at La Trobe University who will be the only person with access to the allocation spreadsheet during the trial. To enrol a participant, the primary researcher (AR) will email the consenting participant’s name and date of birth to the La Trobe University researcher, who will enter the patient into the trial and notify the primary researcher of the treatment group allocation. These details will be entered into the allocation spreadsheet and the next intervention allocation and participant identification number will be emailed to the primary researcher who will contact the treating physiotherapist who will arrange the initial appointment.

### Treatment protocols

The intervention protocols for both groups will be outlined in a detailed treatment manual, supplemented by a digital clinical notes template. The template will detail the intervention in accordance with the study protocol for all sessions. Intervention programs will be matched for time exposure to the physiotherapist, with both groups having six sessions over a 12-week period, at 0, 1, 2, 3, 6- and 12-weeks post randomisation. For both groups the first session will be 1 h in duration with follow-up sessions 30 min.

#### SHOCKWAVE intervention

Intervention in the SHOCKWAVE group will follow the approach of Cacchio et al. [2] consisting of four sessions of ESWT at weekly intervals in accordance with a standardised protocol. There will be no ESWT in the final two sessions, which will be used to review relevant information sheets and plan for return to normal activities.

Although the trial on PHT by Cacchio et al. [2] used only radial shockwave, both radial (EMS Swiss Dolorclast, Milano, Italy) and semi-focused shockwave (Dornier, Germany) will be used as research has found no difference in outcomes between the two different devices in tendinopathy [56]. Shockwave dosage will be 2000 shocks per session at the highest tolerable intensity which appears to be a safe and effective dose [56].

#### PHYSIOTHERAPY intervention

Intervention in the PHYSIOTHERAPY group will relate to known or hypothesised mechanisms underpinning PHT and be informed by treatment shown to be effective in other lower limb tendinopathies [57, 58]. A key component of the program will be a multi-stage, progressive, individualised, strengthening/rehabilitation program, with consideration given to sporting and occupational demands. Graded reintroduction of compressive forces in loading programs is recommended for PHT [3, 59] and other lower limb tendinopathies [60–63] and will be incorporated in the PHYSIOTHERAPY intervention algorithms.

Pain monitoring, both during and latent to loading, is a key component of the intervention. Use of a pain ‘ceiling’ during rehabilitation is thought to provide a safe guideline for exercise loads and avoids the need for a prolonged period of rest in which only pain free activity is allowed. Pain levels of up to 4/10 on a NPRS were permitted, with treating physiotherapist discretion in the presence of higher baseline OMPSQ-SF scores or in the first two repetitions of isometric exercise providing symptoms reduce over this time. A significant increase in symptoms lasting over 24 h after activity is thought to indicate excessive loading of the tendon [3, 14, 64, 65] although the biological mechanism of this response is unknown.

Stage 1 of the PHYSIOTHERAPY intervention will comprise isometric hamstring exercise aiming to safely commence strengthening the hamstring complex, increase motor drive [66] and reduce pain levels. [66]. Stage 2 will incorporate isotonic strengthening exercises of the hamstring musculature. Stage 3 will add kinetic chain exercises including strengthening of agonist muscles, and stage 4 will reintroduce compressive load by increasing the hip flexion angle of hamstring strengthening exercises. The final 5^th^ stage will involve high speed (energy storage and release) exercises included if required for sporting/occupational demands, e.g. field/court sports. Exercises options that are specific to sporting/occupational demands will be chosen where possible. Retraining of lower limb kinetic chain movements (e.g. lunge, squat, running) and lumbopelvic control rehabilitation will be incorporated if indicated by the assessment of the treating physiotherapist in line with recommendations for other lower limb tendinopathies [14, 64]. Progression to later stages of the program will be criteria driven, with demonstrated load tolerance (stable symptoms during, and following, rehabilitation and activity), and strength (compared to the unaffected side) used. Return to sport advice will be provided. The treatment protocols have been developed by the research team including a clinical/research expert in this area (JC) and published elsewhere (manuscript in preparation).

#### Participant education and other co-interventions

Standardised pre-prepared information sheets will be provided to both the SHOCKWAVE and PHYSIOTHERAPY groups. The information sheets will include topics explaining diagnosis, treatment options, expected recovery timeframes, monitoring pain, the role of compression (including sitting) in tendinopathy, and high and low tendon loading activities. Information sheet content will be developed based on known or hypothesised mechanisms underlying physiotherapy treatment of this condition. Management of medication will not be a focus of treatment and will be consistent between groups. Therefore medication use will be measured at baseline only and will not be followed up over time, however physiotherapists will be able to refer participants to a pharmacist or their general practitioner during the treatment program if they determine that medication review is warranted.

#### Participating physiotherapists and treatment fidelity

Physiotherapists from private practices in Victoria will provide treatment for both groups. To be eligible, the physiotherapists will need to have at least 2 years of clinical experience. Physiotherapists will then participate in a small group, 4-h training session provided by the lead researcher (AR). The program will include review of previously provided material, and simulation of explanations and treatments to be used in the trial.

Treating physiotherapists will be provided with a treatment manual detailing treatment algorithms, protocols and participant information sheets. Treatment methods will be clearly defined and standardised via a detailed session-by-session electronic clinical notes template containing a series of decision-making algorithms. The algorithms and clinical notes will ensure that essential elements of the treatment program are consistently applied by all physiotherapists across all participants, while still allowing some opportunity for the treatment to be tailored to individual participant presentation. The template will require treating physiotherapists to provide physical assessment findings, justification and rationale for clinical decision making, detail of treatment provision/prescription and response to treatment. Physiotherapists will be required to complete electronic clinical notes for each session which detail assessment findings, treatment provided, clinical decision-making justification and any adverse events from shockwave treatment or the exercise program.

A quarterly face-to-face meeting will be undertaken for 60 min involving all treating physiotherapists for the duration of the trial to review de-identified cases in the context of the treatment protocol. Evaluation of treatment fidelity and adherence by the physiotherapists for specific rehabilitation techniques will be achieved by checking the physiotherapist’s clinical notes for each participant after the second and fourth sessions of the program.

### Outcome assessment

Outcomes will be assessed through self-administered electronic questionnaires on QuestionPro, except for hamstring strength that will be assessed by a blinded assessor. Weblinks to the questionnaires will be emailed to participants at the appropriate time points (Table 2). Outcome measures adhere to the consensus guidelines for tendinopathy health domains, [67] and patient reporting characteristics (for baseline data) [68]. Feasibility for this trial will be measured with recruitment rate, participant retention, and completion of outcome measures.

**Table 2:** Outcome measures

Outcome measure	Measurement point (weeks)
**Primary outcome measures**	
1. Global rating of change scale (7-point Likert scale)	4, 12, 26, 52
2. VISA-H (8-item questionnaire)	0, 4, 12, 26, 52
**Secondary outcome measures**	
1. Sitting tolerance (5-point scale)	0, 4, 12, 26, 52
2. Modified Physical Activity Level Scale (6-point scale)	0, 4, 12, 26, 52
3. Eccentric hamstring strength (nM)	0, 12
4. Modified Tampa scale for kinesiophobia (TSK-11) (11-item questionnaire)	0, 4, 12, 26, 52
5. Örebro Musculoskeletal Pain Screening Questionnaire Short Form (ÖMPSQ-SF)	0, 4, 12, 26, 52
6. Pain Catastrophizing Scale (PCS)	0, 4, 12, 26, 52
7. Numerical Pain Rating Scale (average and most severe pain over previous week)	0, 4, 12, 26, 52
8. Participant and physiotherapist rating of adherence (11-point Likert scale)	4, 12, 26, 52
9. Satisfaction with treatment (5-point Likert scale)	4, 12, 26, 52
10. Satisfaction with the results of treatment (5-point Likert scale)	4, 12, 26, 52
11. EuroQoL-5D	0, 4, 12, 26, 52

#### Primary outcome measures

Global rating of change will be measured using a 7-point Likert scale, with participants rating their overall change from baseline [69, 70]. Various versions of this scale are considered to be reliable, responsive and valid [70, 71].

Pain, function and sporting activity will be measured with the VISA-H (Victorian Institute of Sport – Hamstring) questionnaire [72]. The VISA-H has been shown to be valid, reliable and responsive for measuring pain, function and sporting activity in people with PHT [72].

#### Secondary outcome measures

Pain with sitting is a common feature of PHT [3] and this will be measured using a Patient Specific Functional Scale (PSFS) [73]. The PSFS is validated and reliable for measuring change with specific functional activities in musculoskeletal conditions [74–78].

Functional restrictions due to the condition will be measured with the modified Physical Activity Level Scale [58, 79] which is validated for measuring physical activity [80]. Eccentric hamstring strength will be measured with the NordBoard (Vald Performance, Albion Queensland) device. Reduced strength has been identified as a risk factor for development of tendinopathy by an expert panel, [81] and many physiotherapy programs incorporate strength exercises. This is a reliable method of testing eccentric knee flexor forces during the Nordic hamstring exercise without provoking symptoms [82].

Three questionnaires will be used to measure psychosocial outcomes. The Orebro Musculoskeletal Pain Screening Questionnaire Short Form (OMPSQ-SF) will be used as an overall measure of psychosocial risk factors. This OMPSQ-SF has been validated for persistent and musculoskeletal conditions, [83] and contains subsections for measuring fear avoidance beliefs, recovery expectations, depression and anxiety. Kinesiophobia will be measured with the Modified Tampa scale (TSK-11) [84]. Kinesiophobia has previously been shown to be associated with some tendinopathies such as rotator cuff [85] but not others, such as lateral elbow tendinopathy [86]. Kinesiophobia appears to be a risk factor for poor outcome in Achilles tendinopathy [87]. The Pain Catastrophizing Scale (PCS) [88] will be used to measure catastrophising. Catastrophising has been shown to be associated with pain and disability in other lower limb tendinopathies [89].

Severity of symptoms will be assessed with the Numerical Pain Rating Scale (NPRS), with participants asked to report their average and worst pain over the preceding week. The NPRS is valid and reliable for musculoskeletal conditions [90].

Participant adherence will be measured using the number of sessions attended, and with participant and physiotherapist report of adherence to treatment [91].

Participants will rate their satisfaction with treatment and their satisfaction with the results of treatment on separate 5-point Likert scales [92–94]. These scales have good reliability, validity and responsiveness [95, 96].

Health-related quality of life will be measured with the EuroQoL-5D, [97] which is valid and responsive in chronic musculoskeletal pain [98].

### Adverse events

Adverse events occurring during the treatment period will be recorded by the physiotherapist in the standardised clinical notes of each participant. Any serious adverse events will be immediately reported to the lead researcher, who will investigate and organise medical care (if required) and report to the ethics committee. The lead researcher will review the clinical notes and questionnaires after 4 weeks of treatment to screen for any unreported adverse effects of treatment. Participants will be provided opportunity on follow-up questionnaires to report any unpleasant, adverse, or harmful effects they ascribe to the treatment.

### Participant adherence and co-interventions

The treating physiotherapist will record the number of treatment sessions attended for each participant, as well as any cancelled or missed appointments. Participant rating of adherence to physiotherapist advice will be recorded formally at each timepoint. Additionally, therapist and participant rating of participant adherence to advice will be recorded at each session. Any co-interventions will be recorded at each follow-up point.

### Data integrity

Outcome data will be stored within QuestionPro and downloaded to an electronic spreadsheet by a research assistant who will be blinded to the group allocation of participants. QuestionPro logs a date-stamp for each questionnaire completed, enabling researchers to monitor completion rates and follow-up missing questionnaires. Data will be reviewed for missing and outlier data to screen for potential data entry errors.

### Blinding

It is not feasible to blind participants or treating physiotherapists due to the nature of the interventions. However, treating practitioners and researchers will inform participants that both treatment approaches have a realistic chance of providing success and that neither has been shown to be superior in previous trials. Physiotherapists will be advised to treat both groups of participants with the same level of expectation and enthusiasm. Strength outcomes will be measured by a blinded assessor. Data analysis will be performed using data without identifying group labels, to ensure blinded analysis.

### Data analysis

#### Sample size

There are no trustworthy data for the VISA-H or other relevant outcome measures for PHT to inform sample size calculations. A sample size of 100 was therefore pragmatically chosen for this pilot study to provide sufficient data to facilitate an accurate sample size calculation and determine the feasibility of future trials on this population. A sample size of 100 would provide 80% power to detect a between-group standardised mean difference of at least 0.6 on continuous outcome measures such as the VISA-H, allowing for a 10% loss to follow up [99]. As smaller effect sizes would still be considered clinically important, this will not be a fully powered trial and is therefore considered a pilot trial.

#### Feasibility

We will aim for a recruitment rate of 12 participants per month. We will aim for greater than 85% participant retention and outcome measures completed, in line with the PEDro scale [100]. A slower recruitment rate would increase the cost and time-commitment of a larger trial.

#### Treatment effects

Following trial completion, data from all follow-up points (4, 12, 26- and 52-weeks following randomisation) will be analysed, focussing on between-group treatment effects (with 95% confidence intervals). SPSS will be used to conduct analyses. Alpha will be set at 0.05 using a two-tailed hypothesis.

Intention to treat principles will be used for all analyses; with participants analysed based on their original allocation regardless of their adherence with treatment or number of sessions attended [101]. Missing data will be managed by maximum likelihood estimation within linear mixed models [102].

Continuous data will be analysed using linear mixed models, adjusting for baseline values and the stratification variable of age (with the group x time interaction estimating the between-group treatment effect). Ordinal data will be analysed using the Mann Whitney U test at each timepoint. It is acknowledged that this will increase the Type 1 error rate however this only applies to some secondary outcomes and is offset in part by the lower power associated with non-parametric tests.

A responder analysis will also be undertaken to determine the proportion of participants who achieved clinically important changes on outcome measures. For these purposes, the minimum clinically important difference (MCID) for individuals will be defined as 12 points on the VISA-H questionnaire [72], and at least ‘much improved’ on the global rating of change scale [103, 104]. The MCID value for the VISA-H was used as it is similar to MCID values on other VISA scales [105–108]. For responder analyses, the risk ratio, risk difference and number needed to treat will be calculated along with 95% confidence intervals [109]. Statistical significance for the responder analyses will be evaluated using Chi square analysis.

## Discussion

In this pilot RCT we aim to compare individualised physiotherapy to ESWT in people with PHT. We hypothesise that participants receiving the individualised physiotherapy program (consisting of a multi-stage, graded strength/rehabilitation exercises) will achieve superior long-term (12 weeks and greater) clinical outcomes to participants who receive ESWT. There may be superior outcomes in the short term (< 12 weeks) in the SHOCKWAVE group due to the analgesic response of ESWT.

We will be testing this hypothesis in a population with longstanding, non-compensable PHT. We are including adult participants with varying levels of sporting participation. This decision was made both to aid recruitment, and to increase generalisability of trial findings. We will avoid including participants with acute or sub-acute PHT as these “reactive” tendinopathies are more likely to settle with short-term reduced load [5] and ESWT may be less likely to be beneficial [110].

To maximise treatment fidelity we will be using strategies similar to those in previous physiotherapy trials [46]. These include a detailed treatment manual, small group training of physiotherapists in the trial, algorithmic clinical note templates, and standardised participant information/advice sheets. Use of multiple stages in rehabilitation and algorithmic treatment approaches allow treatment to be as specific and relevant for participants as possible. As an example, a participant with a goal of returning to tennis may be prescribed lunges as part of their rehabilitation to simulate the demands of volley and backhand strokes. The rehabilitation may progress by modifying joint angles (specifically hip flexion) and eventually by adding speed. In contrast a participant with a goal of returning to walking may not require all of these components of rehabilitation.

While this study lacks a placebo control, it will be of interest to clinicians as it compares two popular treatment options for PHT. We will stratify randomisation by age only and not gender, which may be a limitation in the event that males and females are unbalanced between groups. A limitation of the study is lack of long-term follow-up of strength which could potentially be informative, although other outcomes will be collected at 6 and 12-months. While MRI may have been a helpful addition to the clinical diagnosis used in the study, the cost was prohibitive especially given that imaging is not a gold standard diagnostic tool for PHT.

Results from this pilot trial will be helpful in determining estimates of effect size and variability in outcome data, which will assist to determine sample size calculations and feasibility of future trials. The thresholds for feasibility will be a recruitment rate of at least 12 participants per month and a loss to follow-up of less than 15%. Recruitment for this study started in July 2021, with final recruitment expected to occur in early 2023.

Protocol version 7: Issue date 7 March 2023.

## Supplementary Information

**Additional file 1.** 

## Data Availability

Data will be stored post-project on Research Online, BLINDED’s institutional repository. Access to deidentified data will be available on request to the lead author.
