# Supplementary material for: A pilot randomised trial comparing individualised physiotherapy versus shockwave therapy for proximal hamstring tendinopathy: a protocol
Source: J Exp Orthop. 2023 May 25;10:55. doi: 10.1186/s40634-023-00615-x (PMC10212870; doi:10.1186/s40634-023-00615-x)
Supplement: Supplementary file 1 — Additional file 1. [file 40634_2023_615_MOESM1_ESM.docx]

**Supplementary File 1: Differential diagnosis justification for physical examination screening**

Testing details for other pain-generating structures, with reproduction of reported symptoms considered a positive test

1. Sciatic nerve entrapment: Active piriformis test, Seated piriformis stretch test.(1)
2. Ischiofemoral impingement: Ischiofemoral impingement (IFI) test, long-stride walking (LSW) tests.(2)
3. Hip joint: the Anterior Impingement Test and the Flexion-Abduction-External Rotation Test.(3)
4. Adductor magnus tendinopathy: Prone adductor squeeze with active hip extension.
5. Lumbar spine: Palpation performed with the patient prone, with the examiner applying pressure centrally over the lumbar spinous processes and unilaterally over the lumbar zygapophyseal joints and/or transverse processes (4).

A full neurological examination if indicated from the screening questionnaire (Vroomen et al 2000).

References

1. Martin HD, Kivlan BR, Palmer IJ, et al. Diagnostic accuracy of clinical tests for sciatic nerve entrapment in the gluteal region. *Knee Surg Sports Traumatol Arthrosc*. 2014;22:882-8.

2. Gómez-Hoyos J, Martin RL, Schröder R, et al. Accuracy of 2 Clinical Tests for Ischiofemoral Impingement in Patients With Posterior Hip Pain and Endoscopically Confirmed Diagnosis. *Arthroscopy*. 2016;32:1279-84.

3. Tijssen M, van Cingel REH, de Visser E, et al. Hip joint pathology: relationship between patient history, physical tests, and arthroscopy findings in clinical practice. *Scand J Med Sci Sports*. 2017;27:342-50.

4. Maitland GD. Vertebral Manipulation. Kent: Elsevier Science; 2014.
